# Multimodality matters in numerical communication

**DOI:** 10.3389/fpsyg.2023.1130777

**Published:** 2023-07-26

**Authors:** Bodo Winter, Tyler Marghetis

**Affiliations:** ^1^Department of English Language and Linguistics, University of Birmingham, Birmingham, United Kingdom; ^2^Cognitive and Information Sciences, University of California, Merced, Merced, CA, United States

**Keywords:** numerical cognition, numerical communication, multimodality, data visualization, graphs, gesture, quantifiers

## Abstract

Modern society depends on numerical information, which must be communicated accurately and effectively. Numerical communication is accomplished in different modalities—speech, writing, sign, gesture, graphs, and in naturally occurring settings it almost always involves more than one modality at once. Yet the modalities of numerical communication are often studied in isolation. Here we argue that, to understand and improve numerical communication, we must take seriously this multimodality. We first discuss each modality on its own terms, identifying their commonalities and differences. We then argue that numerical communication is shaped critically by interactions among modalities. We boil down these interactions to four types: one modality can *amplify* the message of another; it can *direct* attention to content from another modality (e.g., using a gesture to guide attention to a relevant aspect of a graph); it can *explain* another modality (e.g., verbally explaining the meaning of an axis in a graph); and it can *reinterpret* a modality (e.g., framing an upwards-oriented trend as a bad outcome). We conclude by discussing how a focus on multimodality raises entirely new research questions about numerical communication.

## 1. Introduction

Humans are wizards of language and number. While honeybees convey to each other the location of flowers, they are not known to buzz around discussing their gustatory benefits. But we humans share elaborate stories about anything and everything. Our immense expressive power makes us unique in the animal world, “the symbolic species” (Deacon, 1997). Another source of human uniqueness is our numerical competence. Many aspects of modern life depend fundamentally on numbers. As Cantlon et al. (2009, p. 83) observe, “In a world without numbers, we would be unable to build a skyscraper, hold a national election, plan a wedding or pay for a chicken at the market.” Construction, politics, social rituals, commerce—each requires not only numbers, but also *talk about numbers*. To hold an election, for instance, votes must be tallied, communicated between election officials, aggregated to a national count, and ultimately conveyed to the public.

This paper provides a new perspective on numerical communication by highlighting its *multimodality*. It is now generally accepted that language is fundamentally multimodal (Kendon, 2004; Perniss, 2018; Holler and Levinson, 2019), spanning not just speech and writing, but also gesture and sign. Most utterances are multimodal, described by scholars as “composite utterances” (Enfield, 2009) or “composite signals” (Clark, 1996). Beyond language, research on communication in a wider sense has long recognized the importance of modalities other than speech or writing (Hull and Nelson, 2005; Finnegan, 2014), especially the visual modality (Kress and Van Leeuwen, 2020), largely due to its importance in print, televised, and internet news. And when it comes to communication about numbers in particular, the visual modality is ubiquitous, from line graphs to bar charts (e.g., Zacks et al., 2002).

Numerical communication is often studied in a unimodal fashion, with different research traditions emphasizing different slices of the multimodal pie. Some psychologists and linguists, for example, have focused on verbal expressions that quantify, such as English “more than half,” “several,” and “few” (Newstead et al., 1987; Moxey and Sanford, 1993b; Coventry et al., 2010; Cummins, 2015). But seldom are these expressions studied in their natural habitat: accompanied by gestures, graphs, or other facets of a multimodal utterance. On the other hand, data visualization researchers have extensively studied how people understand different types of graphs (Cleveland and McGill, 1984, 1985; Zacks and Tversky, 1999; Shah and Hoeffner, 2002; Shah and Freedman, 2011; Padilla et al., 2018; Franconeri, 2021; Franconeri et al., 2021), but experiments in this field generally manipulate visual features, not the accompanying language or gesture.

Isolating specific aspects of numerical communication for study would suffice if naturalistic communication typically involved only one modality at a time. But this is seldom the case. Consider, for example, corporate annual reports. They perform important functions within our economic system. They are subject to legal audits. And they influence stakeholder decisions. These days, one would be hard pressed to find a corporate annual report that does not include graphs (Beattie and Jones, 2001; Frownfelter-Lohrke and Fulkerson, 2001; Guddal, 2016; Nunez, 2016; Kathrada et al., 2021), and at the same time any corporate annual report also contains language that contextualizes and frames what is seen in the graphs. The same applies to most graphs in other contexts—in the news, in academic papers, in the classroom. Rarely do we find a graph without accompanying language.

For numerical communication, multimodality is the rule rather than the exception. Consider, for example, how business presentations frequently use visual aids such as PowerPoint, with possibly millions of visual presentations per day (Parker, 2001). The data communicated visually in these presentations are embedded in verbal narratives. And whenever people give live presentations, they also tend to gesture, for example, by pointing to graphs. The same is true outside the boardroom. Multimodal analyses of TV news, for instance, have found that when news anchors and political commentators discuss facts and figures on the TV, their speech is typically accompanied by numerical gestures (Winter et al., 2013; Woodin et al., 2020; Alcaraz-Carrión et al., 2022). In fact, a look at how numerical concepts have developed over the course of human history suggests that numerical competence has always been interwoven with multimodal means of expression, such as material artifacts used for record keeping (Overmann, 2016, 2018). In naturalistic contexts, therefore, numerical presentations is seldom unimodal. The natural ecology of data-driven decision-making is resolutely multimodal.

Research about *just* language or *just* graphs or *just* sign or *just* gesture risks missing something fundamental about how numerical information is communicated. This paper thus argues for a deeply multimodal approach to studying numerical communication. We first review how different modalities express numerical content (Section 2). We then discuss commonalities across modalities (Section 3), as well as differences (Section 4). Following this, we discuss how the different modalities interact and complement each other such that the whole multimodal utterance is more than the sum of its unimodal parts (Section 5), which we then demonstrate with an extended example from an expert communicator (Section 6).

## 2. Modalities of numerical communication

### 2.1. Overview

Communication occurs in different modalities. Consider speech and gesture: one communicates *via* sound, the other *via* shape and motion. But modalities can differ not just in medium of expression, but also mode of representation (Chrisomalis, 2020, p. 150; Hull and Nelson, 2005). From this perspective, a written expression such as “1.2 million” is multimodal because it combines different modes of representing numerical information: a written number word that is specific to English (“million”), and a largely translinguistic numeral (“1.2”; Chrisomalis, 2020, Ch. 6).

Each modality offers a variety of strategies for communicating numerical information. In the following, we structure our discussion of these strategies in terms of approximate versus exact quantity. People are able to think about numbers both approximately and exactly (Dehaene, 1997; Feigenson et al., 2004). While exact and approximate quantity may not be represented and processed by distinct cognitive systems (Klein et al., 2009; Cheyette and Piantadosi, 2020), we use this contrast here to capture the range of goals and strategies that are evident in numerical communication. We communicate approximate magnitudes. And we communicate exact numerical values. Different modalities offer a range of ways to accomplish both of these communicative goals.

### 2.2. Exact numerical communication

**Figure 1:**
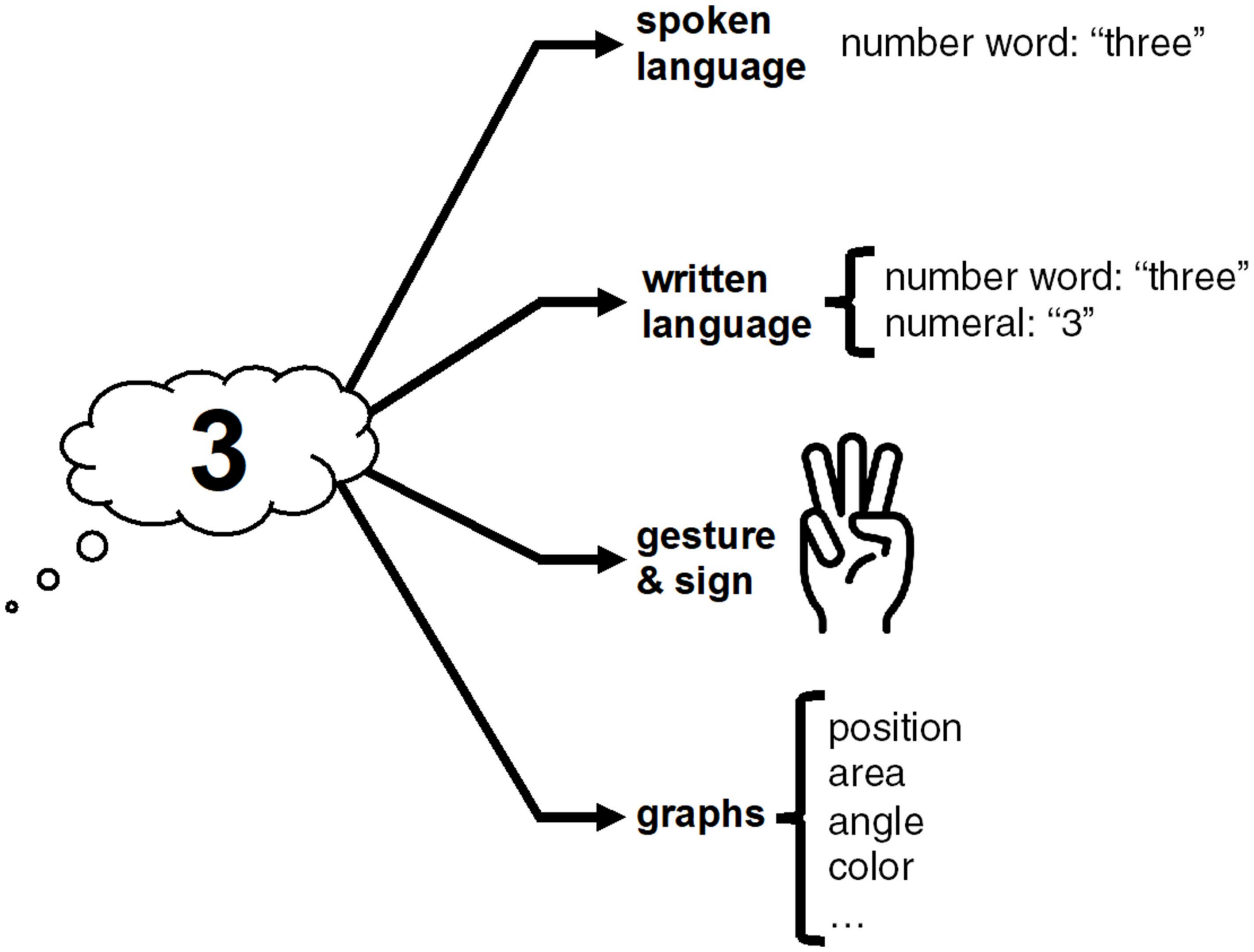
Multiple modalities to express exact numerals; compare Chrisomalis (2020, p. 151).

Exact numbers can be expressed *via* spoken language, written language, gesture, and graphs, as shown in Figure 1, inspired by Chrisomalis (2020, p. 151). In English, the number three, for example, can be expressed *via* a spoken or written number word (“three”), or *via* a written numeral (“3”), or *via* gesture (e.g., three extended fingers), or *via* various visual features of graphs, such as the height of a bar in a bar chart or color in a heat map. We will discuss each representational modality in turn.

First, number words: The majority of numeral systems across the world’s spoken languages use a base-10 ‘decimal’ system (Comrie, 2013), but there exist important cross-linguistic differences (Hurford, 1975). For example, French has a mixed decimal-vigesimal system, with some numbers expressed as multiples of 20 (e.g., 80 is ‘quatre-vingts,’ *four-twenties*). Languages also differ in *how many* numbers can be expressed precisely. The Pirahã language spoken in the Amazon, for example, totally lacks words for exact numbers, making it difficult to refer verbally to exact numerical values (Frank et al., 2008; see also Butterworth et al., 2008).

Signed languages also vary in how they express exact number. To communicate exact values, signed languages typically use the fingers (Ulrike et al., 2013). Like spoken languages, many signed languages use a decimal system, including British Sign Language, American Sign Language, and German Sign Language. But, also like spoken languages, there are important cross-cultural differences, for example, the Mardin Sign Language of Turkey, has—similar to spoken French—a vigesimal subsystem where 40, 60, and 80 are expressed as multiples of 20 by flicking 2, 3, or 4 fingers, respectively (Ulrike et al., 2013). Mardin Sign Language also has a subtractive subsystem where numbers such as 19 and 18 are formed as the equivalent of ‘twenty one-less’ and ‘twenty two-less.’ Languages both spoken and signed, therefore, typically include subsystems for expressing exact numbers using language-specific rules.

Another modality for expressing exact number is written notation, which can follow rules that differ from those for spoken or signed language. Although the history of numerical notation is entangled with the history of writing, numerical notations have their own cultural trajectory (Chrisomalis, 2020). For instance, what are commonly known as the ‘Arabic’ numerals (1, 2, 3, …) are used today by a wide range of language communities, including speakers of languages from unrelated families. But Arabic numerals are not universal, and they can co-exist with notational systems such as the Chinese (一, 二, 三) and Roman (I, II, III; Chrisomalis, 2010).

Yet another modality is gesture, a universal aspect of face-to-face communication (McNeill, 1992; Kendon, 2004). The hands can represent exact quantities by holding the fingers in particular configurations (e.g., lifting two fingers to indicate “two”), a practice known as “finger montring” (Di Luca and Pesenti, 2008) or “number gestures” (Gibson et al., 2019). Even though this involves the “natural” artifact of our fingers, this practice is nevertheless cultural, with considerable cross-cultural diversity (Bender and Beller, 2012). Even within closely connected Western cultures there can be differences, such as between Germans, who gesture “3” with the thumb, index finger, and middle finger, as opposed to members of Anglo-Saxon cultures, who are more likely to use the index, middle, and ring finger. This cultural difference was popularized in Quentin Tarantino’s World War II film *Inglourious Basterds,* where Allied spies are discovered when they order “three beers” with a non-German numerical gesture.

Finally, one of the dominant ways exact numbers are expressed in industrialized societies is *via* graphs. Within a graph, numerical values can be represented by a variety of spatial and aesthetic dimensions. For example, numbers can be mapped to a color gradient (e.g., 1 represented by yellow, 3 represented by orange, 5 represented by red), or to the angle of a pie chart, or the area of mosaic plot, or the *x*- and *y*-coordinates of a point or line. The specific visual features chosen by a graph designer to represent numerical values have consequences for communication. For example, it can be harder to make exact numerical comparisons using pie charts compared to the height of bars (e.g., Cleveland and McGill, 1984; Simkin and Hastie, 1987), although in some contexts, pie charts may be advantageous (e.g., Peterson and Schramm, 1954; Hollands and Spence, 2001; Kosara, 2019b). Thus, choices about how, specifically, to encode numerical values in graphs have implications for how people interpret the values, with downstream consequences for data-driven decisions (Zacks and Franconeri, 2020).

### 2.3. Conveying approximate numerical information… using exact numerical values

We now move from exact to approximate numerical communication. The strategies available to convey approximate quantities are so extensive that it is impossible to draw a neat picture like Figure 1. To put some structure to our discussion, we distinguish between utterances that convey approximate numerical information using expressions that involve exact numerical values (Section 2.2), and utterances that do not mention exact numerical values at all (Section 2.4).

One strategy for expressing approximate quantities using exact numerical values is *rounding*. Even though the expression, “There were 3000 people at the protest,” involves a phrase which literally denotes an exact value (“three thousand”), this expression is typically *interpreted* as expressing a rounded value (Krifka, 2007; Cummins, 2015; Solt et al., 2017) with a certain amount of pragmatic slack (Lasersohn, 1999). The exact value is used to express a range of allowable values. If the number of people at the protest was exactly 2,999, 2,998, 3,001, or 3,002, stating that there were “3,000 people” would typically be accepted as speaking truthfully (*cf.* Krifka, 2007). Thus, even though round numbers *can* denote exact values, they can also be used to express approximate quantities. Rounded values are used disproportionally in both spoken and written language (Dehaene and Mehler, 1992; Coupland, 2011; Woodin et al., 2023).

People use rounding for a variety of reasons. One is to maximize communicative relevance and minimize cognitive difficulty. English speakers will give inexact numbers that adhere to salient landmarks when telling time, e.g., “quarter to four” rather than “three forty-two,” even if they know the exact time, presumably because the exact time is deemed irrelevant for the listener (Van Der Henst et al., 2002; Gibbs and Bryant, 2008), or because a rounded number is easier to comprehend and remember than its exact counterpart (Solt et al., 2017; Nguyen et al., 2022). People may also report rounded numbers because they are actually uncertain about the exact quantity (Ruud et al., 2014), or instead, even if they know the exact quantity, they may prefer to not report it so as to not appear pedantic (Beltrama et al., 2022). The use of exact values for approximate ends also allows for strategic manipulation (e.g., Cummins and Franke, 2021), as when *p*-values in academic publications are rounded up or down to make research results appear as ‘significant’ as possible (Krawczyk, 2015; Hartgerink et al., 2016).

Another strategy that uses exact numbers for approximate purposes is numerical hyperbole (Lavric, 2010), such as when saying, “It took a million years to get to the table,” which is unlikely to be interpreted literally (Kao et al., 2014). In numerical hyperbole, the literal sense of a phrase is too unlikely to be taken seriously, and the phrase is thus interpreted as referring to *some* exceptionally large or small numerical value.

Rounding and numerical hyperbole do not exhaust the range of approximate expressions in English that feature exact values in their construction (Channell, 1994, Ch. 3). Other examples include the “*n* or *m*” construction, as in “it costs three or four bucks,” or the “*n* or so” construction, as in “ten pounds or so.” Numerical values can also be combined with approximators (“about 1,000”) or bounded quantifiers (“at least 1,000,” “more than 1,000”; Channell, 1994; Cummins, 2015). In informal contexts, even more expressions are available, such as “3,000 give or take,” “ballpark 3,000,” “3,000-ish,” and many others (Ferson et al., 2015).

The approximate use of exact numerical values also appears in graphs. While graphs are typically generated such that they represent numbers exactly, the actual *use* of graphs is typically approximate. First, although our visual system can take in an astonishing amount of information simultaneously, there is a limit to vision’s numerical precision. Moreover, our visual system may be subject to illusions and biases that distort the numerical information presented, leading to over- or under-estimation of numerical values in particular types of graphs (e.g., Xiong et al., 2020a). This, too, means that even though graphs may be accurate *in production*, they may involve a considerable degree of deviation from exact values *in perception*.

The approximate perception of graphs is seldom a problem, however, since the goal of a graph is seldom to communicate exact values but rather to emphasize rough comparisons (Washburne, 1927). A line graph can convey whether there is an increasing or decreasing trend. Or a bar chart can convey whether one group is bigger than another. In fact, graphs are rarely used in decision making contexts in which it is necessary to retrieve exact values (see review in Kosslyn, 2006, p. 31), and if precision really matters, tables of exact values may be preferred instead. The goal of graphs is usually to convey overall, gross patterns. Thus, even though computerized graphs involve the representation of exact values, their actual use—both perceptually and functionally—involves a considerable degree of approximation.

**Figure 2:**
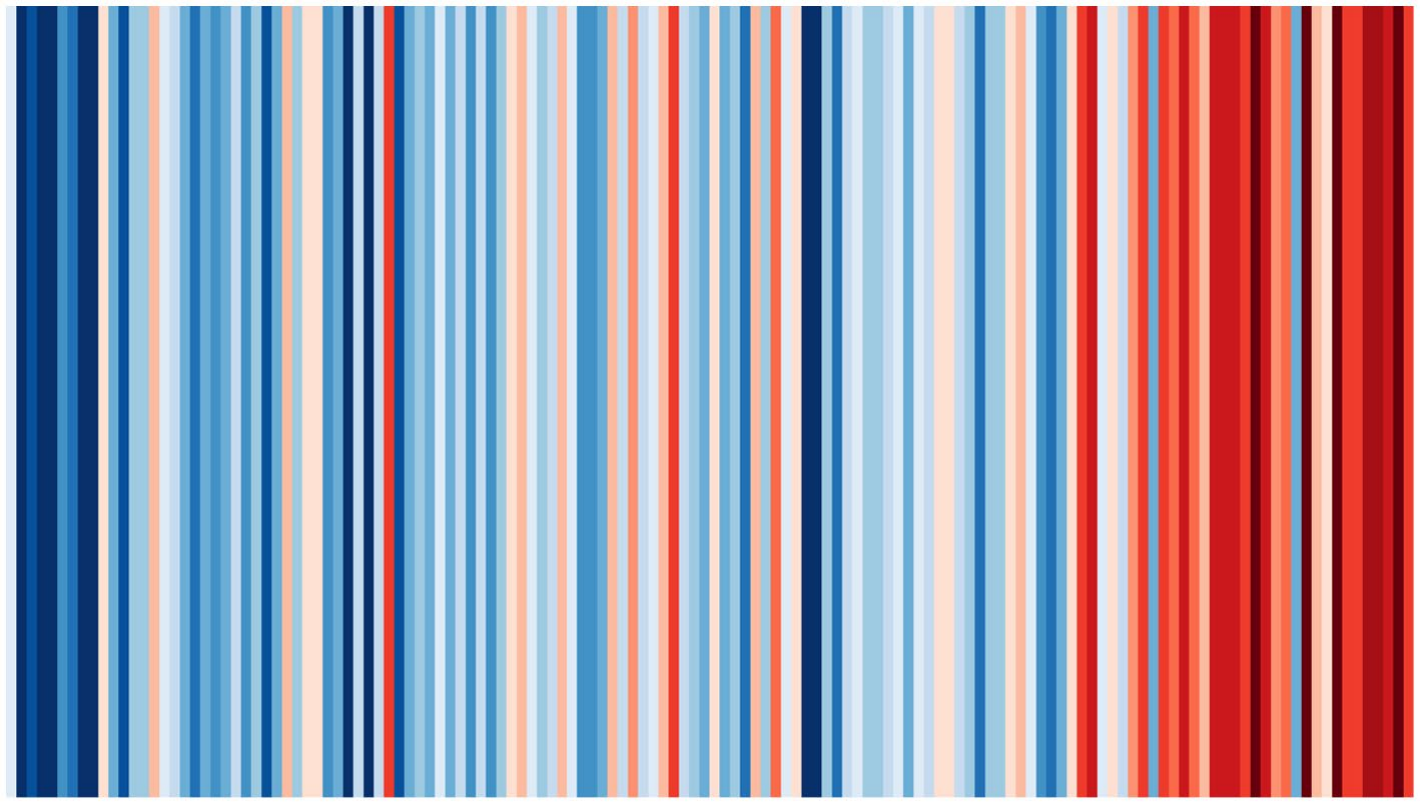
Visualization of temperature over time (1884–2021) for England, United Kingdom, using data from the United Kingdom Met Office. The use of color to convey numerical information encourages approximate rather than exact comparison (This #ShowYourStripes image by Ed Hawkins is licensed under CC BY 4.0. Available at: https://showyourstripes.info/).

A great example of a data visualization that is clearly intended to de-emphasize exact values is the #ShowYourStripes visualization, originally conceived by Ed Hawkins. Figure 2 is an example of such a chart, showing average temperature data for England, United Kingdom, from 1884 to 2021. Horizontal position encodes time and color encodes average temperature in this graph. People on Twitter were motivated to produce similar visualizations for their location using the hashtag #ShowYourStripes to increase climate change awareness. Given the absence of any numerical values in the version shown in Figure 2, this graph is ill-suited to convey precise values. Instead the message is approximate and holistic: the globe is getting hotter.

### 2.4. Approximate numerical communication without exact values

In addition to utterances that use exact values to convey inexact quantities, there are a whole range of approximate expressions that involve no mention of numerical values. This includes vague quantifiers such as “many,” “a lot,” “few,” “several,” which have been at the center of research in formal semantics, experimental pragmatics, and psychology (for reviews, see Moxey and Sanford, 1993a; Cummins, 2015). There are also expressions involving nouns, such as “loads of,” “the majority of,” “masses of,” and “oodles of” (Channell, 1994, Ch. 5), and even fictitious numbers such as “umpteen,” “gazillion” or “squillion” (Rips, 2013; Chrisomalis, 2016).

An especially interesting class of expressions used to convey approximate quantities are those involving spatial language. In English, there are two dominant ways in which this can be done, either using vertical or size-based language (Lakoff and Johnson, 1980; Lakoff and Núñez, 2000; see also Núñez and Marghetis, 2015 and Winter et al., 2013). For example, English speakers can refer to numbers as being “high” or “low,” and they can use relative vertical location for comparisons (“this number is lower than that one”). Many verbs that express vertical change can also be co-opted to refer to changing quantities, such as when numbers are described as “rising,” “falling,” “skyrocketing,” “ascending,” “plummeting,” or “soaring” (Smiley et al., 2016). The other set of English expressions uses size-based language, such as when referring to static quantities as “large,” “small,” “tiny” etc., or when referring to changing quantities as “expanding,” “growing,” or “shrinking.”

Importantly, both vertical and size-based ways of *talking* about quantities have analogs in *thinking* about quantities (for review, see Winter et al., 2015a). For example, when people have to press buttons to verify whether a number is even or odd, they are faster to respond to larger numbers when the button is in a high position, and faster to respond to smaller numbers when the button is in a low position (Hartmann et al., 2014). Similarly, when people see quantities of objects (e.g., dots), they estimate that there are more objects when they are spread over a larger region of space, thus confounding numerical quantity and spatial extent (Hurewitz et al., 2006). Thus, the ways English speakers talk spatially about quantity have parallels in how people think spatially about quantity in nonlinguistic tasks.

The presence of these vertical and size-based mappings in numerical *thinking* may account for why the same mappings show up not just in conventional language but also in spontaneous co-speech gestures. Earlier work found that gestures that convey numerical information are widespread in technical discourse among expert mathematicians (Núñez, 2004, 2008; Marghetis and Núñez, 2013). More recently, Woodin et al. (2020) demonstrated that such gestures show up widely in public discourse. They investigated how American English speakers describe numerical information on television, looking at data from more than 500 speakers using the phrases “tiny number,” “small number,” “big number,” and “huge number,” in a sample drawn from the TV News Archive.^1^ Woodin and colleagues found the majority of the time that the speaker was visible and their hands were not occupied, they also produced gestures that were semantically congruent with the implied quantity, such as pinching the index finger and thumb together when talking about a “tiny number,” or extending the hands outwards away from the torso when talking about a “huge number.” These findings closely mirror what has previously been found in laboratory settings, where people automatically respond to smaller numbers with smaller grip apertures (Badets et al., 2007, 2012; Lindemann et al., 2007; Gabay et al., 2013; Grade et al., 2017).

Alcaraz-Carrión et al. (2022) performed a similar analysis of TV news data, looking at over 400 gestures co-occurring with language that involves addition or subtraction in both concrete and abstract contexts, such as “add hundreds of jobs,” “4 plus 5,” or “subtract Trump’s personality.” They found that most utterances about arithmetic were accompanied by gestures, with around 80% of subtraction-related expressions and 60% of addition-related expressions accompanied by co-speech gesture. Gestures tended to move toward the right for addition-related concepts, while those co-occurring with subtraction-related concepts moved toward the left. This closely mimics experimental results on mental arithmetic and attentional biases, which have found that addition shifts spatial attention rightward and subtraction shifts spatial attention leftward (McCrink et al., 2007; Pinhas and Fischer, 2008; Knops et al., 2009; Marghetis et al., 2014).

Importantly, the gestures studied by Woodin et al. (2020) and Alcaraz-Carrión et al. (2022) are approximate, in stark contrast to ‘montring’ three fingers to represent exactly three beers. While the speakers in these contexts may know the exact value they are talking about, the upwards- or outwards-moving gestures that co-occur with such phrases as “high number” or “huge number” are not intended to be interpreted precisely, i.e., it is clear to the onlooker that the overall direction of movement matters, and values are not mapped onto gesture space in a metric fashion (see discussion in Woodin et al., 2020). Thus, gesture is one of the prime outlets through which we communicate numerical information approximately.

These studies are amongst the first to demonstrate that gesture is ubiquitous in public discourse about numerical information. But do people actually pay attention to these gestures? What do they contribute to comprehension? These questions are ripe for future inquiry. So far, studies of whether gestures are functionally relevant for numerical thinking have been largely limited to pedagogical contexts. There is an extensive body of research demonstrating that gestures produced by both students and teachers aid the acquisition of mathematical concepts (e.g., Alibali and DiRusso, 1999; Goldin-Meadow et al., 2001, 2009; Cook et al., 2008, 2013; Alibali and Nathan, 2012; Goldin-Meadow, 2014; Novack and Goldin-Meadow, 2015; Gibson et al., 2019). Compared to the large body of work on numerical gestures in educational contexts, however, there is comparatively little research on the functional relevance of gesture in adult-to-adult communication between numerate individuals (for an exception, see Nicol and Patson, 2022).

## 3. Commonalities across modalities

### 3.1. Overview

Modalities of numerical communication share commonalities. In this section, we discuss the following ‘big-picture’ commonalities:

1. Exact versus approximate expression.
2. Shared cognitive mappings.
3. Shared semiotic principles.

### 3.2. Exact versus approximate expression

As reviewed in Section 2, it is possible to express numerical concepts either exactly or approximately. We can use exact linguistic expressions (“seven”) or approximate ones (“about ten”), as well exact gestures (showing seven fingers) or approximate ones (pinching the fingers together to signal a small quantity). Data visualization may be the exception to this, since it typically involves *exact production* unless drawn by hand, but graph designers can manipulate the degree to which users are cued into paying attention to exact values. For example, graph designers can use an inner grid if precise values are important (Kosslyn, 2006, p. 71), since grids facilitate tracing locations in a scatter plot or height in a bar plot to the corresponding values along the *x*- or *y*-axes. The density of the reference grid can also give cues as to how much precision is implied. Similarly, the fact that the #ShowYourStripes visualization in Figure 2 has no explicit *x*- and *y*-axes labels is a clear sign to the user of the graph that they should not attempt to read off precise values. Across modalities of numerical communication, it is possible to convey both exact and approximate numerical information.

Numbers often have an aura of exactness and objectivity (Porter, 1996). It may thus be surprising that no matter the modality, so much of numerical communication involves approximation rather than exactitude. The relative prevalence of approximate versus exact reference may vary across modalities, though we suspect that the overwhelming majority of naturally occurring numerical communication may be approximate. For example, as mentioned above, even when speakers know precise times, they tend to report times with rounded values (Van Der Henst et al., 2002; Gibbs and Bryant, 2008; Solt et al., 2017). Discourse analyses also support the idea that vague references to quantity dominate even in contexts where we might expect a need for precision, such as academic and scientific discourse (Banks, 1998; Cutting, 2012), business contexts (Koester, 2007), and political discourse in the TV news (Woodin et al., 2020; Alcaraz-Carrión et al., 2022). Moreover, the fact that languages such as English offer rich repertoires of variegated expressions for approximate numerical reference suggests that there is communicative need, as generally languages tend to have more expressions for more common topics or concepts (e.g., Regier et al., 2016; Winter et al., 2018). And, as discussed above, we often use exact visualizations of numerical information in a way that, functionally, is only approximate. Thus, the over-representation of approximate versus exact numerical communication may be another commonality across modalities, on top of the more basic fact that both exact and approximate quantities *can*, in principle, be communicated in all modalities.

### 3.3. Shared cognitive mappings

Mappings between space and number are another commonality across domains. English speakers talk and write about numbers as “rising,” and they also often gesture upwards when using such language (Winter et al., 2013). This mapping between numbers and vertical space can also be found in graphs, where the *y*-axis typically represents higher values with higher vertical locations (see, e.g., Woodin et al., 2022). Size-based mappings of quantity are similarly expressed in language, sign, gesture, and graphs: both language and gesture can represent numerical concepts in terms of spatial extent (e.g., “tiny number” or pinching the fingers together), and data visualizations can represent magnitudes in terms of area, such as in the case of pie charts and bar charts, for which area is the primary perceptual cue for quantity (Skau and Kosara, 2016; Kosara, 2019a). When data visualizations violate these shared cognitive mappings between space and quantity, they are less likely to be understood (e.g., Pandey et al., 2015; Woodin et al., 2022). From this we can form the prediction, so far untested, that verticality-based encodings of quantity in graphs and gesture should benefit from language that uses vertical expressions for quantity, while size-based encodings in graphs and gesture should benefit from size-based language.

Interestingly, not every spatial mapping is attested for all modalities. The horizontal mental number line is a well-established cognitive phenomenon whereby people in Western cultures associate smaller quantities with left space, and associate larger quantities with right space (e.g., Dehaene et al., 1993; Wood et al., 2008). This mapping commonly features in data visualization, with quantities increasing left-to-right along the *x*-axis (e.g., Tversky, 2011), and may also occur in horizontally oriented gestures (Winter et al., 2013; Alcaraz-Carrión et al., 2022; but see Woodin et al., 2020). However, speakers seldom *talk* about numbers in terms of horizontal position. While English speakers might describe a smaller quantity as “low” or “small,” it would be extremely unusual to call it a “left number.” A similar lacuna exists for talk about the domain of *time*, where a left–right mapping appears in thought (a mental timeline), in material artifacts (e.g., calendars), in graphs (e.g., line graphs), and in gesture (e.g., rightward gestures when talking about the future) but is almost entirely absent from *speech*. The one known exception is the variety of English spoken within the US Military, where left–right mappings for time are conventionalized (e.g., “move the meeting two days to the right” to describe a delay; Hendricks et al., 2018). Why certain mappings are only available in some modalities of expression needs to be investigated more closely.

### 3.4. Shared semiotic strategies

Multimodal utterances convey meaning in a variety of ways, exhibiting “semiotic diversity” (Kendon, 2014; see also Ferrara and Hodge, 2018). Within semiotics, a key question is how the form of a sign in the most general sense (such as a word, a manual sign of a signed language, a gesture, or a graph) relates to its meaning. That is, how do we come to know the meaning of a sign from its form?

Following Peirce (1955), a classic taxonomy distinguishes three ways the form of a sign can relate to its meaning: by being *iconic*, *indexical*, or *symbolic.* Iconic signs are those which partially resemble their meanings, such as when the distance between index finger and thumb (form) is used to indicate a small size (meaning; Hassemer and Winter, 2018). Indexical signs are those that involve a direct spatial, temporal or causal connection, such as is the case with pointing gestures, which ‘index’ things in the environment by evoking a vector to the intended referent (Kita, 2003; Hassemer and McCleary, 2018). Finally, ‘symbols’ involve no direct connection between form and meaning. In this case, the meaning can only be accessed by knowing that a particular form conventionally represents a specific meaning. That is, for ‘symbols,’ we do not rely on indexical or iconic cues to infer meaning (Keller, 1998); we merely know that X means Y because we have learned this relationship. In general, these different strategies for conveying meaning are available across modalities (Ferrara and Hodge, 2018), such that we can and often do communicate iconically, indexically, and symbolically regardless of whether we are talking, signing, gesturing, or using graphs.

In the following, we give examples of iconic, indexical, and symbolic signs in the context of numerical communication. Iconicity can be a feature of notational writing systems (e.g., Chinese 一, 二, 三 and ancient Roman numerals I, II, III involve a direct resemblance between form and meaning) as well as of finger counting systems, where form (the number of fingers) mimics meaning (the implied quantity; *cf.* Bender and Beller, 2012). Iconicity is an inherent feature of almost all graphs since there is almost always a direct resemblance relation between visual form features and their intended quantitative meanings, for example, higher vertical locations indicate larger quantities. Iconicity is also a feature of many gestures, such as when the height of a vertical gesture indicates the denoted quantity. And iconicity can be a feature of numerical communication in speech as well. People raise their voice pitch when talking about smaller objects (Perlman et al., 2015), and this pitch-based iconicity can be co-opted for talk of numerical quantities, such as when speaking of a “tiiiiiiiny quantity” with raised voice pitch. There is very little research on iconicity in spoken ways of expressing numerical information (but see Coulter and Coulter, 2010), perhaps because iconicity for numerical quantity is overall more common in the graphical and gestural domains.

Indexicality, too, plays a role in all modalities of numerical communication. When presenting a graph, for example, we can refer to specific aspects of the display by using a pointing gesture, or adding an arrow to the graph, or uttering an indexical expression such as “this bar over here.” Clark (1996, 2003) discusses indexicality as the act of directing the interlocutor’s attention, and when viewed from this perspective, any aspect of a graph that directs attention to certain features, such as salient colors that draw attention to themselves, can be seen as having an indexical component. Indeed, a common recommendation by guides for data visualization is to use visual elements such as color to highlight relevant data patterns (Ajani et al., 2022). Focusing can also be done *via* other modalities, such as gesture, sign, and speech, and not just elements that are intrinsic to the graph itself. Directing attention is thus an important aspect of communicating numerical information—especially when the information is abundant or complex, straining a recipient’s capacity to make sense of it.

Numerical communication also relies crucially on the final semiotic mode of communication, the ‘symbolic.’ Number words and quantifiers are conventional expressions; we generally need not rely on iconicity or indexicality to understand them. Moreover, even the rampant iconicity of graphs relies on graphical conventions for precise interpretation. Researchers have thus argued for the importance of considering graph literacy or ‘graphicacy’ in education (Balchin, 1972; Fry, 1981). The fact that graphs are not always understood correctly without explicit instruction demonstrates the importance of these graphical conventions. Graphs in Cartesian coordinates, for instance, use distance from the origin to represent numerical values—thus using distance *iconically*—but people must learn the graphical *convention* that numerical values increase upward and rightward, not downward and leftward (Woodin et al., 2022). Even richly iconic graphs can thus include symbolic elements.

## 4. Each modality has its own vibe

### 4.1. Overview

While there are important similarities shared across modalities, there are also important differences. Here, we focus on the following four dimensions of difference:

1. Sequential versus simultaneous presentation.
2. Permanence.
3. Reliance on expertise.
4. Plausible deniability.

### 4.2. Sequential versus simultaneous presentation

Modalities differ in whether information is presented sequentially or simultaneously. Spoken language, for instance, requires that we transform a complex message into a sequence of words, one after the other (Hull and Nelson, 2005, p. 229). Gestures have elements of sequentiality in that language users can string together sequences of gestures, but individual gestures often exhibit simultaneity, too. For example, one can say “five plus three” while producing a bimanual ‘collecting’ gesture in which each hand represents a different addend and their inward motion simultaneously represents their addition (Núñez and Marghetis, 2015). Such a gesture simultaneously expresses two values (i.e., the two hands) and the mathematical operation of addition (the inward movement), thus conveying three elements at the same time. In comparison, the phrase “five plus three” conveys the same three elements sequentially.

Graphical representations that are static and not animated can convey even more information simultaneously. Indeed, one of the unique advantages of graphs is that they can convey multiple pieces of information at the same time, thus communicating patterns that can be processed in parallel by the viewer (e.g., Zacks and Franconeri, 2020). Take, for example, the verbal description of a numerical difference between two groups: “this group has a much higher output than the other one.” While this utterance presents information about two quantities in sequence, a graph such as a bar chart could communicate the same information simultaneously, at an instant.

### 4.3. Permanence

Modalities differ in timescale. Speech, sign, and gesture are typically transient: a spoken word is gone as soon as it is spoken; a sign or gesture must be seen as it is being produced, since just like speech, it typically leaves no record. By contrast, graphical representations are generally more lasting (Latour, 1986): they may last for minutes (e.g., quick whiteboard sketches) or years (e.g., graphs in a published book).

The different components of a multimodal numerical utterance, therefore, will differ in their permanence. One consequence is that when modalities depend on each other for their interpretation, a difference in permanence can have serious consequences for whether the message remains interpretable. A poorly labeled graphical representation may be easily understood if accompanied by a flurry of speech and gesture; but since the graphical representation will likely persist beyond the end of any accompanying speech and gesture, others may encounter it and find it incomprehensible. This misalignment of timescales, therefore, has consequences for how robust a message may be—that is, for how likely the meaning of the message is to degrade over time, even if some modalities of the message remain.

### 4.4. Reliance on expertise

Modalities differ in how expertise with each modality is distributed among the general public. Almost all adults in a language community will know and understand their language’s count list (“one, two, three…”), expressions such as “large number” and “increasing prices,” and accompanying gestures. But graphical literacy is much more dependent on explicit training and expertise (Balchin, 1972; Fry, 1981), and hence something that is also more variable in the population (e.g., Shah and Hoeffner, 2002; Okan et al., 2012).

A reliance on expertise increases the potential for misunderstanding. To make matters worse, experts may assume that their graphical representations stand on their own, easily understood by any intelligent adult (Xiong et al., 2020b). Falling prey to such a “curse of knowledge” or “curse of expertise” has been a point of critique, for example, in the case of the United Kingdom covid press briefings, where government officials showed graphs and used phrases such as “As you can see,” when in fact the majority of the public did not readily see anything in the hastily shown graphs (Cheshire, 2020).

Here again, modalities can complement each other: a presenter can use gestures to point to specific aspects of the graph, or use speech to talk up a graph, highlighting and explaining different components of it. This shows how the effects of low graph literacy can be alleviated by multimodality. In sum, modalities differ in how much they rely on expertise and education.

### 4.5. Plausible deniability: “That’s not what I said…”

Modalities differ in the degree to which they afford plausible deniability and with this, the degree to which they are routinely held to standards of accountability in public discourse. Gesture in particular has been shown to have the power to sway interpretation without being noticed. This has been explored in studies on the “gestural misinformation effect” (Gurney et al., 2013, 2016; Kirk et al., 2015; see also Broaders and Goldin-Meadow, 2010), which demonstrates that when people are asked about an event they saw, such as a crime scene, gestures co-occurring with the question can bias responses in investigative interviews or eye witness reports. For example, people may report a murder weapon that was not actually part of the scene they saw, but that was primed *via* a gesture. What is particularly troublesome about this finding is that in contrast to verbal information, the influence of gesture often goes unnoticed, even when participants are warned about its potentially biasing effects (Haynes, 2017).

Gestures thus have the ability to influence interpretation and decision making in a way that often bypasses the standards of accountability that we usually have for verbal language. This can be exploited by politicians, who often choose their words carefully to avoid making controversial statements, but who can use gestures strategically as part of persuasive moves. For example, Hart and Winter (2021) performed a qualitative analysis of the populist politician Nigel Farage who speaks of an “explosion” or “sheer volume” of immigrants entering the United Kingdom, and while doing so, uses expansive size gestures to exaggerate the number of immigrants involved. These gestures form part of his campaign ads and are watched by millions of people. Yet the transcript of *speech* is typically taken to be the official record of “what was said,” erasing the contributions of gesture. Since co-speech gestures inform what was *understood,* even if not put into words, gesture offers the potential for unnoticed influence.

## 5. Putting together the pieces

### 5.1. Emerging meaning in multimodal messages

How, then, do different modalities combine in a multimodal utterance? One possibility is that they combine additively, like a layer cake, with each modality adding another independent layer of information to the holistic cake of communication. On this account, the communicative impact of each individual modality does not depend on any others. Speech, for instance, would add information completely independently of whatever is happening concurrently in gesture or writing. The fact that numerical communication is multimodal, on this account, is important only because each modality offers more or different information, but the contribution of each modality could be understood on its own. In other words, the presence of one modality does not fundamentally transform the contributions of any of the others. If multimodality was additive in this way, then it would suffice to study each modality on its own.

But multimodality is *not* additive. Each component of multimodal communication shapes the others; the meaning of the whole is more than the sum of its parts (Hull and Nelson, 2005). In other words, multimodal utterances are interaction-dominant. Their meaning reflects not just the sum of the unimodal parts, but their cross-modal interactions. This is neatly demonstrated by co-speech gesture, which can disambiguate and sometimes completely reverse the interpretation of a message. A number of studies have shown this using the following ambiguous question: “Next Wednesday’s meeting has been moved forward 2 days, what day is it on now?” Without seeing a concomitant gesture, about half of all respondents report that the meeting has been moved to “Friday,” and half report it has been moved to “Monday” (McGlone and Harding, 1998; Lewis and Stickles, 2017). This ambiguity in language can be resolved *via* gesture—for instance, a forward gesture that points toward the future. Indeed, if the speaker uses such a forward gesture, the majority of people think the meeting is now on Friday. But this pattern reverses if the speaker gestures backward toward their torso, after which most people think the meeting has been moved to Monday (Jamalian and Tversky, 2012; Lewis and Stickles, 2017; Winter and Duffy, 2020).

The interaction-dominant rather than additive nature of multimodality is also demonstrated by how the meaning of gestures is underdetermined (Kendon, 2004; McNeill, 2005). Calbris (2011) describes gestures as “polysemous,” the same way that words are polysemous when they have multiple different meanings depending on context. Take, for example, a person tracing an upward and rightward trajectory with an extended index finger. This could be intended to direct one’s attention to a crack on the wall, or to enact a driving route on an imagined map. On its own, this gesture—like any gesture—is ambiguous, open to a wide range of interpretations, many of which may not be numerical or mathematical in nature. Accompanied by speech, however, the meaning of this gesture can become numerical. Gestures exactly like this one, for instance, are regularly produced by mathematicians when describing technical mathematical concepts such as “continuous functions” or “increasing values” (Núñez, 2004; Marghetis and Núñez, 2013).

### 5.2. Types of interactions

Individual modalities interact in a variety of ways: by amplifying, by directing, by explaining, and by reinterpreting. These are not mutually exclusive, and one multimodal message can simultaneously tap into multiple types of interactions.

The first type of interaction we call amplification: several modalities may *amplify* the same message, that is, the same core idea can be expressed in multiple modalities at the same time. Such amplification can make use of the fact that there are shared mappings between modalities (Section 3.3). For example, in a live presentation, speech, gesture, and graph can simultaneously express an upwards trend, e.g., “a high number of cases,” with a vertically oriented graph in the background and an upwards-sweeping gesture. Importantly, due to the differences between modalities outlined in Section 4, such redundancy always involves diversity as well. Expressing functions redundantly with diverse components has been argued to increase the robustness of systems in general (Kitano, 2004), including the robustness of transmitting messages in communication (Winter, 2014; Mason et al., 2015). Borkin et al. (2016) annotated graphs for ‘data redundancy’ (if the data is visually encoded in more than one way) and ‘message redundancy’ (if the main message of the graph is signaled in more than one way) and showed that both types of redundancy boosted people’s memorization of the graph’s content. Given the differences between modalities, it is often impossible to encode the same information in exactly the same way, so modalities are seldom truly “redundant.” We thus speak of “amplification,” with the same information expressed in different, complementary ways across multiple modalities.

There are multiple reasons why it may be beneficial to multimodally amplify the same information. First, encoding something into memory *via* multiple representational formats, such as verbal and visual ones, leads to better recall (Paivio, 1971, 1986). Second, different perceivers may have different modality-specific preferences, e.g., paying more attention to visual or verbal content. A message that is encoded simultaneously both visually and verbally is therefore more likely to stick with more people. Third, differences between modalities (Section 4) can be used to make up for each individual modality’s shortcomings. For example, if somebody does not look at the speaker while they perform a gesture, they may still hear the corresponding verbal phrase (“a high number of cases”) or be able to make sense of the message by looking at a corresponding graph. From this perspective, there are enough commonalities for the modalities to work in tandem (e.g., shared mappings), but enough differences to complement each other (e.g., differences in permanence). To conclude, amplifying is more than just redundantly expressing the same content in different modalities. Amplification increases robustness of numerical communication by encoding the same content *differently*.

The second type of interaction involves using one modality to *direct* the receiver’s attention to a particular aspect of a multimodal display. The power of one modality to direct attention to another is exemplified by the foundational cultural practice of *finger counting*, in which the cardinality of a set of objects is determined by coordinating speech (“one, two, three …”) with gesture (pointing to each item in a sequence, or lifting one finger at a time; Alibali and DiRusso, 1999). While the gestures produced during finger counting do not typically represent exact number on their own, they direct attention toward the physical environment in coordination with exact expressions in speech. The practice works holistically because one modality directs attention while another keeps track of exact quantity.

As another example, consider the typical graphical representation consisting of various parts—points, bars, lines, labels—many of which may be inessential to the speaker’s primary message. Gesture and speech can direct attention to the graph’s essential aspects—a recent increase, for instance, or a relative difference between two data points. This emphasis can have huge implications for the holistic message, such as when a confusing sea of points in a scatterplot is winnowed down by speech and gesture to two essential points that differ in unexpected ways (“here [pointing gesture] and here [pointing gesture]”). Modalities can thus interact to create a holistic message not by changing the meaning of a graph, but by changing the focus or attentional spotlight.

As discussed in Section 4.2, graphs afford more simultaneous expression of information than speech, gesture, or signs. However, some graphs can involve so much information that they cannot be comprehended at a glance, requiring viewers to engage in a sequential comparisons between different points in the graph (Nothelfer and Franconeri, 2020). As discussed in relation to the curse of knowledge (Section 4.4), the most relevant components of a graph may be obvious to the designer but not the audience (Xiong et al., 2020b). For complex visual displays, therefore, the directing function of speech and gesture becomes especially important.

A third type of interaction involves using one modality to *explain* the content of another modality, e.g., unpacking some aspect that would be otherwise opaque or confusing. For example, the axes of a graph may have terse labels that are difficult to interpret when first encountered, but that may make sense once explained. Here, coupling the graphic with speech and gesture (e.g., pointing to one axis and saying, “And here we have the net profits after accounting for depreciation”) can unpack the inner workings of the graphical representation.

Finally, one modality can *reinterpret* another modality, transforming its meaning from one interpretation to an entirely different one. A graph showing an upward trend, for example, might be interpreted as good news (Woodin et al., 2022), but this default interpretation could be inverted by accompanying speech, e.g., “disastrous rise” (see also discussion in Franconeri, 2021, Figure 5). In instances of multimodal reinterpretation, therefore, the meaning of one modality is transformed by the rest of the multimodal message.

Whether amplifying, directing, explaining, or reinterpreting, cross-modal interactions are shaped by the unique affordances of each modality. For example, we have discussed how graphs are relatively less transient (Section 4.3) and more simultaneous (Section 4.2). Skilled communicators combine modalities in ways that are informed by these cross-modal differences. For instance, good presenters talk up a graph by combining language and gesture to help the audience understand a graph’s meaning. The graph’s permanence can then be used to anchor more transient gestures and verbal statements, such as when multimodal utterances highlight specific sub-components (“Here on the x-axis, you see …”). The sequentiality of speech and gesture allow the presenter to impose a sequential interpretation, thus helping viewers make sense of graphical information in a piecemeal fashion. Thus, it is the differences between modalities that make them so effective in combination.

Table 1 gives a schematic overview of the dimensions of multimodality discussed in this paper, including commonalities (left column), differences (middle column), and interactions (right column).

**Table 1:** Modalities and their commonalities (left), differences (middle) and interactions (right).

Commonalities	Differences	Interactions
(1) Exact versus approximate expression	(1) Sequential versus simultaneous presentation	(1) Amplifying
(2) Shared cognitive mappings	(2) Permanence	(2) Directing
(3) Shared semiotic principles	(3) Reliance on expertise	(3) Explaining
	(4) Plausible deniability	(4) Reinterpreting

## 6. Tapping into the power of multimodality: a case study of expert numerical communication

We want to conclude with an example demonstrating the combination of multiple modalities by an expert communicator. Wingspan Productions teamed up with the Swedish physician and public health specialist Hans Rosling to create a show for BBC Four called *The Joy of Stats*, which included a notable sequence where Rosling described large-scale geopolitical patterns in wealth and health over the last two centuries. We focus our analysis on the opening section, for which we highlight all four types of interaction between modalities (*cf.* Section 5). In a single sequence of multimodal utterances, Rosling demonstrates how modalities can interact by *explaining* and *directing* (Section 6.1), *reinterpreting* (Section 6.2), and *amplifying* (Section 6.3).

### 6.1. Explaining and directing

**Figure 3:**
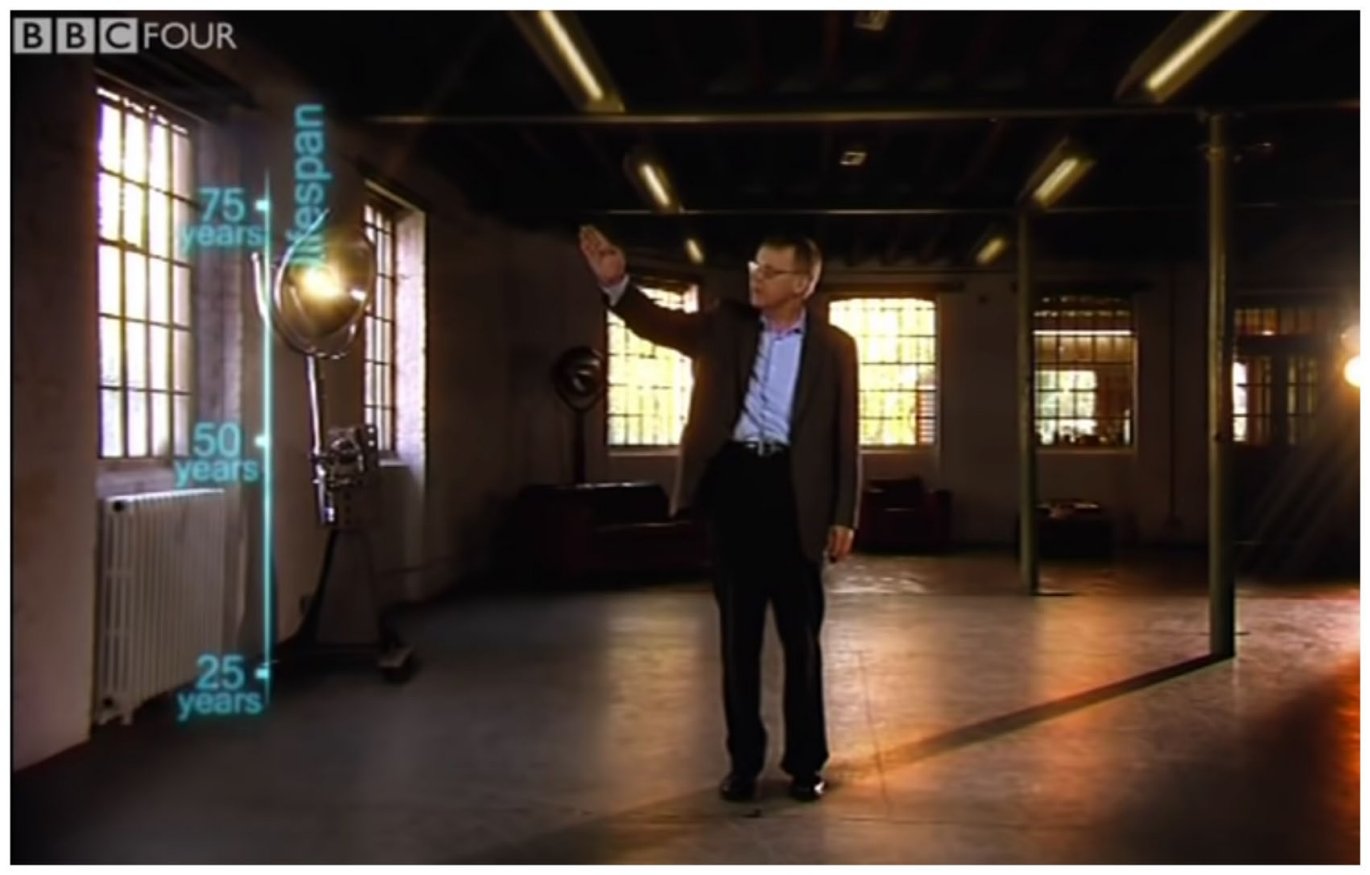
Still image of Hans Rosling in the BBC Four *The Joy of Stats* pointing toward the *y*-axis; video available at: https://www.youtube.com/watch?v=jbkSRLYSojo (Accessed December 21, 2022). Reproduced with the permission of Wingspan Productions Ltd.

Hans Rosling begins his explanation with a gesture, pointing to his right while saying: “First an axis for health: life expectancy from 25 to 75 years” (Figure 3). This multimodal utterance is accompanied by an animation: a line for the y-axis that grows from bottom to top, thereby conveying the directionality of the axis (younger to older), something lacking from a static image. Tick marks along the axis appear in an animated sequence of three steps, one for each tick mark, from shortest lifespan (25 years) to longest lifespan (75 years). This reinforces the upwards directionality of this axis.

**Figure 4:**
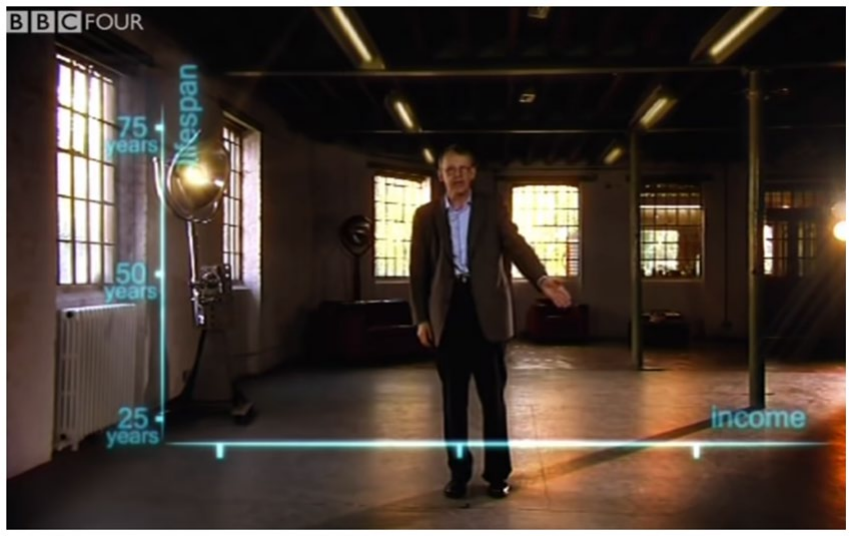
Final position of the sweeping gesture, which started by pointing to the origin and then swept along the *x*-axis. Reproduced with the permission of Wingspan Productions Ltd.

Rosling then introduces the *x*-axis with his left hand sweeping along the bottom of the emerging graph, from the viewer’s left to the viewer’s right (see Figure 4). He accompanies this gesture by the verbal expression, “And down here, an axis for wealth; income per person, 400, 4000, and 40,000 dollars.” His gesture and speech thus combine to both orient the viewer’s attention (“down here”) and to explain the directionality of the axis. While a reader typically needs to know the convention to interpret the directionality of a static graph’s axes, here Rosling combines speech, gesture, and animation to alleviate the need for audience expertise.

**Figure 5:**
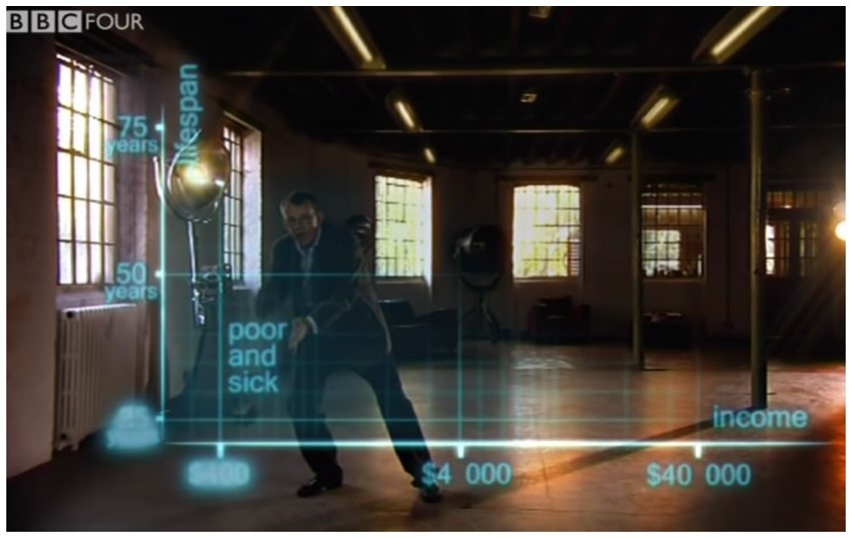
The lower-left quadrant is highlighted in three different ways: via speech (“down here”), a two-handed pointing gesture, and via tick marks becoming highlighted. Reproduced with the permission of Wingspan Productions Ltd.

Rosling then adds an explanation: “so, down here is poor and sick.” While doing this, he moves his whole body to the lower quadrant of the superimposed graph and points to the corner with two hands (Figure 5). In addition, the tick marks associated with the lower left quadrant briefly flare up, further highlighting the lower left quadrant *via* graph-internal means. The entire multimodal display thus combines to focus the viewer’s attention, with speech (“down here”), gesture, and graphical display all exhibiting indexicality.

### 6.2. Reinterpreting

Rosling completes his introduction of the graph by describing the upper right quadrant as “rich and healthy.” Throughout the sequence, the vertical axis is described as “health” but actually represents a much more specific quantity, life expectancy. The horizontal axis is described as “wealth,” but represents income. His accompanying speech thus encourages a more general, big-picture framing of specific quantities. One modality thus encourages *reinterpretation* of another, and the entire multimodal utterance is consequently reframed in terms of more general issues.

### 6.3. Amplifying

**Figure 6:**
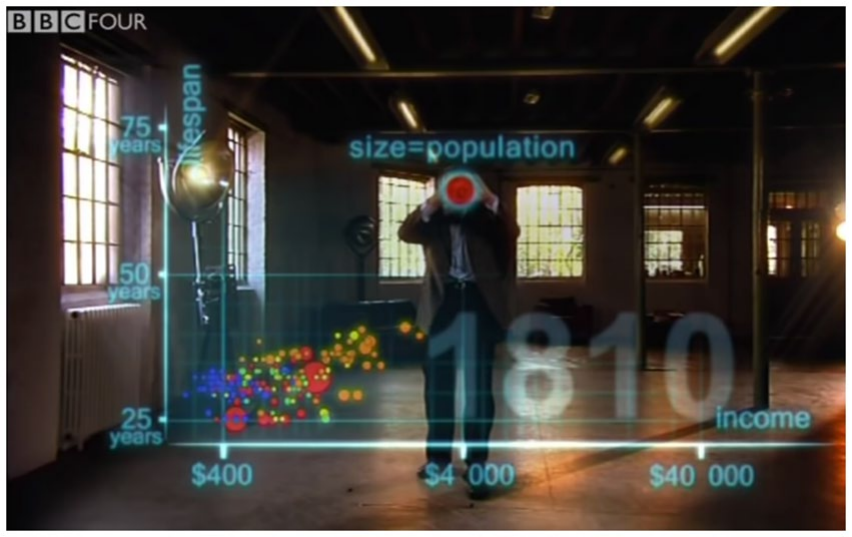
Speech, gesture, and graphical display are combined to convey a conceptual mapping between quantity and area. Hans Rosling uses speech to explain how the size of each data point represents population size, while simultaneously forming a circle with two hands. An animated circle is superimposed on his hands, along with written text (“size=population”). Reproduced with the permission of Wingspan Productions Ltd.

After adding data points for the individual countries, Rosling then says, “the size of the country bubble showed the size of the population,” while simultaneously using a two-handed gesture that forms a circle (see Figure 6). At the same time an animated red circle is superimposed onto the gesture. The fact that circle size represents population size is reiterated in the written modality *via* a superimposed text that reads “size = population,” displayed just above the red circle and gesture. Four aspects thus combine to communicate the mapping between population and area: speech, gesture, superimposed circle, and superimposed text. The same mapping is expressed in an amplified fashion *via* simultaneous expression in different modalities.

## 7. Conclusion

Multimodality matters in numerical communication. Much is lost if we consider only one modality at a time—studying graphs without language, or language and graphs without gesture. This is because naturally occurring numerical communication is typically multimodal. One is hard pressed to find examples of purely unimodal communication of numerical information. And this ubiquitous multimodality has implications for how and what we communicate. The meaning of numerical communications reflects the ways modalities can interact, with one modality able to amplify, direct, explain, or reinterpret the others. As a result, the meaning of a multimodal display of numerical information is more than the sum of its parts.

A multimodal perspective on numerical communication invites new research questions. For example, one could look at the effectiveness of indexicality across modalities, such as whether highlighting relevant data patterns within larger graphical displays works better or worse with graph-internal means (e.g., arrows, salient colors) or graph-external means (e.g., gesture and speech). Similarly, many topics already being researched in the data visualization literature have natural multimodal analogs. For example, many graph design guides for practitioners mention how Gestalt principles can be used to form natural groupings of elements, but gesture, too, can be used to group elements together, as has been explored in the context of grouping addends for students in explaining math equations (e.g., Goldin-Meadow et al., 2009).

Likewise, there is a lot of existing research on how graphs can convey the *uncertainty* of numerical information (e.g., Padilla et al., 2021). This, too, could be studied multimodally. For example, speakers can use expressions such as “perhaps” and “probably,” or numerical hedge words that express approximation “about” or “give or take” to communicate uncertainty in information verbally (Ferson et al., 2015). But they can also use gestures to achieve the same effect, for instance referring to an exact quantity while holding both hands outwards in a ‘shrug’ type gesture, palms facing upwards to express uncertainty (e.g., Cooperrider et al., 2018). We do not know how these modalities interact when communicating uncertainty about numerical information. Much of the ways in which speech, gesture, sign, and graphs interact in communicating numerical content is uncharted territory. The field is ripe with opportunities for new discoveries.

Multimodal communication also presents novel opportunities for the study of numerical cognition, including freely available multimodal data such as the TV News Archive (used in Woodin et al., 2020) or the Little Red Hen project (used in Alcaraz-Carrión et al., 2022). These provide new means of testing ideas that could previously only be assessed in less ecologically valid laboratory settings. For example, people have found interactions between numbers and space along the horizontal, vertical, and sagittal (front-back) axes (Winter et al., 2015b). Recent work suggests that all three axes may play a role at the same time (Aleotti et al., 2020, 2022), which makes a clear prediction for when people speak about large numerical quantities: speakers should be more likely to gesture to their right, front, and upwards when referring to larger quantities. As gesture is a medium of expression that naturally lives in three dimensions, it provides an ideal means for looking at the co-activation of multiple spatial representations, as has been shown for mental associations between space and time (Walker and Cooperrider, 2016). Woodin et al. (2020) discuss some of the interconnections between numerical cognition research and multimodal datasets.

Understanding the multimodality of numerical communication is not merely of academic interest. It has immediate real-world implications—for best practices in clear communication, for the dangers of distortion, and for the accessibility of messages. Crafting effective messages requires awareness and strategic use of multimodality. For instance, guides for practitioners of data visualization often focus on low-level aspects of the visual system. We call for future guides to consider more strongly not just the graphical displays themselves, but the language and gestures that surround them. Indeed, effectively conveying a data-driven message to decision-makers depends on more than the clarity of a graph in isolation, but also the accompanying language and gestures that help avoid misunderstandings and encourage insights. As exemplified by Hans Rosling’s discussion of the interplay of health and wealth, the best numerical communicators are richly and creatively multimodal, taking advantage of the opportunities afforded by each modality and their interactions.

## Author contributions

All authors listed have made a substantial, direct, and intellectual contribution to the work and approved it for publication.

## Funding

BW was supported by the UKRI Future Leaders Fellowship MR/T040505/1.

## Conflict of interest

The authors declare that the research was conducted in the absence of any commercial or financial relationships that could be construed as a potential conflict of interest.

## Publisher’s note

All claims expressed in this article are solely those of the authors and do not necessarily represent those of their affiliated organizations, or those of the publisher, the editors and the reviewers. Any product that may be evaluated in this article, or claim that may be made by its manufacturer, is not guaranteed or endorsed by the publisher.

## Footnotes

^1^ https://archive.org/details/tv

## References

[ref1] Ajani K. Lee E. Xiong C. Knaflic C. N. Kemper W. Franconeri S. (2022). Declutter and focus: empirically evaluating design guidelines for effective data communication. IEEE Trans. Vis. Comput. Graph. 28, 3351–3364. doi: 10.1109/TVCG.2021.3068337, PMID: 33760737

[ref2] Alcaraz-Carrión D. Alibali M. W. Valenzuela J. (2022). Adding and subtracting by hand: metaphorical representations of arithmetic in spontaneous co-speech gestures. Acta Psychol. 228:103624. doi: 10.1016/j.actpsy.2022.103624, PMID: 35667244

[ref3] Aleotti S. Di Girolamo F. Massaccesi S. Priftis K. (2020). Numbers around Descartes: a preregistered study on the three-dimensional SNARC effect. Cognition 195:104111. doi: 10.1016/j.cognition.2019.104111, PMID: 31731115

[ref4] Aleotti S. Massaccesi S. Priftis K. (2022). The SNARC effect: a preregistered study on the interaction of horizontal, vertical, and sagittal spatial–numerical associations. Psychol. Res. 87, 1256–1266. doi: 10.1007/s00426-022-01721-8, PMID: 35960336 PMC10191909

[ref5] Alibali M. W. DiRusso A. A. (1999). The function of gesture in learning to count: more than keeping track. Cogn. Dev. 14, 37–56. doi: 10.1016/S0885-2014(99)80017-3

[ref6] Alibali M. W. Nathan M. J. (2012). Embodiment in mathematics teaching and learning: evidence from learners’ and teachers’ gestures. J. Learn. Sci. 21, 247–286. doi: 10.1080/10508406.2011.611446

[ref7] Badets A. Andres M. Di Luca S. Pesenti M. (2007). Number magnitude potentiates action judgements. Exp. Brain Res. 180, 525–534. doi: 10.1007/s00221-007-0870-y, PMID: 17279382

[ref8] Badets A. Bouquet C. A. Ric F. Pesenti M. (2012). Number generation bias after action observation. Exp. Brain Res. 221, 43–49. doi: 10.1007/s00221-012-3145-1, PMID: 22744775

[ref9] Balchin W. G. V. (1972). Graphicacy. Geography 57, 185–195.

[ref10] Banks D. (1998). Vague quantification in the scientific journal article. ASp. La Revue Du GERAS 19–22, 17–27. doi: 10.4000/asp.2666

[ref11] Beattie V. Jones M. J. (2001). A six-country comparison of the use of graphs in annual reports. Int. J. Account. 36, 195–222. doi: 10.1016/S0020-7063(01)00094-2

[ref12] Beltrama A. Solt S. Burnett H. (2022). Context, precision, and social perception: a sociopragmatic study. Lang. Soc. 1–31, 1–31. doi: 10.1017/S0047404522000240

[ref13] Bender A. Beller S. (2012). Nature and culture of finger counting: diversity and representational effects of an embodied cognitive tool. Cognition 124, 156–182. doi: 10.1016/j.cognition.2012.05.005, PMID: 22695379

[ref14] Borkin M. A. Bylinskii Z. Kim N. W. Bainbridge C. M. Yeh C. S. Borkin D.. (2016). Beyond memorability: visualization recognition and recall. IEEE Trans. Vis. Comput. Graph. 22, 519–528. doi: 10.1109/TVCG.2015.2467732, PMID: 26390488

[ref15] Broaders S. C. Goldin-Meadow S. (2010). Truth is at hand: how gesture adds information during investigative interviews. Psychol. Sci. 21, 623–628. doi: 10.1177/0956797610366082, PMID: 20483837 PMC2902555

[ref16] Butterworth B. Reeve R. Reynolds F. Lloyd D. (2008). Numerical thought with and without words: evidence from indigenous Australian children. Proc. Natl. Acad. Sci. 105, 13179–13184. doi: 10.1073/pnas.0806045105, PMID: 18757729 PMC2527348

[ref17] Calbris G. (2011). Elements of Meaning in Gesture (Vol. 5). Amsterdam: John Benjamins Publishing.

[ref18] Cantlon J. F. Platt M. L. Brannon E. M. (2009). Beyond the number domain. Trends Cogn. Sci. 13, 83–91. doi: 10.1016/j.tics.2008.11.007, PMID: 19131268 PMC2709421

[ref19] Channell J. M. (1994). Vague Language. Oxford, UK: Oxford University Press.

[ref20] Cheshire J. (2020). Next slide please: data visualisation expert on what’s wrong with the UK government’s coronavirus charts. *The Conversation*. Available at: http://theconversation.com/next-slide-please-data-visualisation-expert-on-whats-wrong-with-the-uk-governments-coronavirus-charts-149329

[ref21] Cheyette S. J. Piantadosi S. T. (2020). A unified account of numerosity perception. Nat. Hum. Behav. 4, 1265–1272. doi: 10.1038/s41562-020-00946-0 32929205

[ref22] Chrisomalis S. (2010). Numerical Notation: A Comparative History. Cambridge, UK: Cambridge University Press.

[ref23] Chrisomalis S. (2016). Umpteen reflections on indefinite hyperbolic numerals. Am. Speech 91, 3–33. doi: 10.1215/00031283-3509480

[ref24] Chrisomalis S. (2020). Reckonings: Numerals, Cognition, and History. Cambridge, MA: MIT Press.

[ref25] Clark H. H. (1996). Using Language. Cambridge, UK: Cambridge University Press.

[ref26] Clark H. H. (2003). “Pointing and placing” in Pointing: Where Language, Culture, and Cognition Meet. ed. Kita S. (Mahwah, NJ: Lawrence Erlbaum), 251–276.

[ref27] Cleveland W. S. McGill R. (1984). Graphical perception: theory, experimentation, and application to the development of graphical methods. J. Am. Stat. Assoc. 79, 531–554. doi: 10.1080/01621459.1984.10478080

[ref28] Cleveland W. S. McGill R. (1985). Graphical perception and graphical methods for analyzing scientific data. Science 229, 828–833. doi: 10.1126/science.229.4716.828, PMID: 17777913

[ref29] Comrie B. (2013). “Numeral Bases” in The World Atlas of Language Structures Online. eds. Dryer M. S. Haspelmath M. (Leipzig, Germany: Max Planck Institute for Evolutionary Anthropology)

[ref30] Cook S. W. Duffy R. G. Fenn K. M. (2013). Consolidation and transfer of learning after observing hand gesture. Child Dev. 84, 1863–1871. doi: 10.1111/cdev.12097, PMID: 23551027

[ref31] Cook S. W. Mitchell Z. Goldin-Meadow S. (2008). Gesturing makes learning last. Cognition 106, 1047–1058. doi: 10.1016/j.cognition.2007.04.010, PMID: 17560971 PMC2265003

[ref32] Cooperrider K. Abner N. Goldin-Meadow S. (2018). The palm-up puzzle: meanings and origins of a widespread form in gesture and sign. Front. Commun. 3:23. doi: 10.3389/Fcomm

[ref33] Coulter K. S. Coulter R. A. (2010). Small sounds, big deals: phonetic symbolism effects in pricing. J. Consum. Res. 37, 315–328. doi: 10.1086/651241

[ref34] Coupland N. (2011). How frequent are numbers? Lang. Commun. 31, 27–37. doi: 10.1016/j.langcom.2010.09.001

[ref35] Coventry K. R. Cangelosi A. Newstead S. E. Bugmann D. (2010). Talking about quantities in space: vague quantifiers, context and similarity. Lang. Cogn. 2, 221–241. doi: 10.1515/langcog.2010.009

[ref36] Cummins C. (2015). Constraints on numerical expressions (Vol. 5). Oxford, UK: Oxford University Press.

[ref37] Cummins C. Franke M. (2021). Rational interpretation of numerical quantity in argumentative contexts. Front. Commun. 6, 1–16. doi: 10.3389/fcomm.2021.662027

[ref38] Cutting D. J. (2012). Vague language in conference abstracts. J. Engl. Acad. Purp. 11, 283–293. doi: 10.1016/j.jeap.2012.05.004

[ref39] Deacon T. W. (1997). The Symbolic Species: The Coevolution of Language and the Brain. New York, NY: W. W. Norton and Company.

[ref40] Dehaene S. (1997). The Number Sense. Oxford University Press, New York City.

[ref41] Dehaene S. Bossini S. Giraux P. (1993). The mental representation of parity and number magnitude. J. Exp. Psychol. Gen. 122, 371–396. doi: 10.1037/0096-3445.122.3.371

[ref42] Dehaene S. Mehler J. (1992). Cross-linguistic regularities in the frequency of number words. Cognition 43, 1–29. doi: 10.1016/0010-0277(92)90030-L, PMID: 1591901

[ref43] Di Luca S. Pesenti M. (2008). Masked priming effect with canonical finger numeral configurations. Exp. Brain Res. 185, 27–39. doi: 10.1007/s00221-007-1132-8, PMID: 17909768

[ref44] Enfield N. J. (2009). The Anatomy of Meaning: Speech, Gesture, and Composite Utterances (Vol. 8). Cambridge, UK: Cambridge University Press.

[ref45] Feigenson L. Dehaene S. Spelke E. (2004). Core systems of number. Trends Cogn. Sci. 8, 307–314. doi: 10.1016/j.tics.2004.05.002 15242690

[ref46] Ferrara L. Hodge G. (2018). Language as description, indication, and depiction. Front. Psychol. 9, 1–15. doi: 10.3389/fpsyg.2018.00716, PMID: 29875712 PMC5974176

[ref47] Ferson S. O’Rawe J. Antonenko A. Siegrist J. Mickley J. Luhmann C. C.. (2015). Natural language of uncertainty: numeric hedge words. Int. J. Approx. Reason. 57, 19–39. doi: 10.1016/j.ijar.2014.11.003

[ref48] Finnegan R. (2014). Communicating: The Multiple Modes of Human Communication. 2nd Edn. Milton Park: Routledge.

[ref49] Franconeri S. L. (2021). Three perceptual tools for seeing and understanding visualized data. Curr. Dir. Psychol. Sci. 30, 367–375. doi: 10.1177/09637214211009512

[ref50] Franconeri S. L. Padilla L. M. Shah P. Zacks J. M. Hullman J. (2021). The science of visual data communication: what works. Psychol. Sci. Public Interest 22, 110–161. doi: 10.1177/15291006211051956 34907835

[ref51] Frank M. C. Everett D. L. Fedorenko E. Gibson E. (2008). Number as a cognitive technology: evidence from Pirahã language and cognition. Cognition 108, 819–824. doi: 10.1016/j.cognition.2008.04.007, PMID: 18547557

[ref52] Frownfelter-Lohrke C. Fulkerson C. L. (2001). The incidence and quality of graphics in annual reports: an international comparison. J. Bus. Commun. 38, 337–357. doi: 10.1177/002194360103800308

[ref53] Fry E. (1981). Graphical literacy. J. Read. 24, 383–389.

[ref54] Gabay S. Leibovich T. Henik A. Gronau N. (2013). Size before numbers: conceptual size primes numerical value. Cognition 129, 18–23. doi: 10.1016/j.cognition.2013.06.001, PMID: 23811178

[ref55] Gibbs R. W. Jr. Bryant G. A. (2008). Striving for optimal relevance when answering questions. Cognition 106, 345–369. doi: 10.1016/j.cognition.2007.02.008, PMID: 17433280

[ref56] Gibson D. J. Gunderson E. A. Spaepen E. Levine S. C. Goldin-Meadow S. (2019). Number gestures predict learning of number words. Dev. Sci. 22:e12791. doi: 10.1111/desc.12791, PMID: 30566755 PMC6470030

[ref57] Goldin-Meadow S. (2014). How gesture works to change our minds. Trends Neurosci Educ 3, 4–6. doi: 10.1016/j.tine.2014.01.002, PMID: 25396115 PMC4225621

[ref58] Goldin-Meadow S. Cook S. W. Mitchell Z. A. (2009). Gesturing gives children new ideas about math. Psychol. Sci. 20, 267–272. doi: 10.1111/j.1467-9280.2009.02297.x, PMID: 19222810 PMC2750886

[ref59] Goldin-Meadow S. Nusbaum H. Kelly S. D. Wagner S. (2001). Explaining math: gesturing lightens the load. Psychol. Sci. 12, 516–522. doi: 10.1111/1467-9280.00395, PMID: 11760141

[ref60] Grade S. Badets A. Pesenti M. (2017). Influence of finger and mouth action observation on random number generation: an instance of embodied cognition for abstract concepts. Psychol. Res. 81, 538–548. doi: 10.1007/s00426-016-0760-7, PMID: 26927471

[ref61] Guddal T. (2016). Graph usage in annual reports, evidence from Norwegian listed companies. MA thesis. Nova School of Business and Economics.

[ref62] Gurney D. J. Ellis L. R. Vardon-Hynard E. (2016). The saliency of gestural misinformation in the perception of a violent crime. Psychol. Crime Law 22, 651–665. doi: 10.1080/1068316X.2016.1174860

[ref63] Gurney D. J. Pine K. J. Wiseman R. (2013). The gestural misinformation effect: skewing eyewitness testimony through gesture. Am. J. Psychol. 126, 301–314. doi: 10.5406/amerjpsyc.126.3.0301, PMID: 24027944

[ref64] Hart C. Winter B. (2021). Gesture and legitimation in the anti-immigration discourse of Nigel Farage. Discourse Soc. 33, 34–55. doi: 10.1177/09579265211048560

[ref65] Hartgerink C. H. J. Aert R. C. M. Van Nuijten M. B. Wicherts J. M. Assen M. A. L. M. Van. (2016). Distributions of p-values smaller than 0.05 in psychology: what is going on? PeerJ, 4,:e1935. doi: 10.7717/peerj.1935, PMID: 27077017 PMC4830257

[ref66] Hartmann M. Gashaj V. Stahnke A. Mast F. W. (2014). There is more than “more is up”: hand and foot responses reverse the vertical association of number magnitudes. J. Exp. Psychol. Hum. Percept. Perform. 40, 1401–1414. doi: 10.1037/a0036686, PMID: 24749934

[ref67] Hassemer J. McCleary L. (2018). The multidimensionality of pointing. Gesture 17, 417–463. doi: 10.1075/gest.17018.has

[ref68] Hassemer J. Winter B. (2018). Decoding gestural iconicity. Cogn. Sci. 42, 3034–3049. doi: 10.1111/cogs.12680, PMID: 30471078

[ref69] Haynes B. (2017). Please ignore those gestures: Does warning reduce the gestural misinformation effect? Honors thesis. King’s University College at Western University.

[ref70] Hendricks R. K. Bergen B. K. Marghetis T. (2018). Do metaphors move from mind to mouth? Evidence from a new system of linguistic metaphors for time. Cogn. Sci. 42, 2950–2975. doi: 10.1111/cogs.12693, PMID: 30328150

[ref71] Hollands J. G. Spence I. (2001). The discrimination of graphical elements. Appl. Cogn. Psychol. 15, 413–431. doi: 10.1002/acp.714

[ref72] Holler J. Levinson S. C. (2019). Multimodal language processing in human communication. Trends Cogn. Sci. 23, 639–652. doi: 10.1016/j.tics.2019.05.006, PMID: 31235320

[ref73] Hull G. A. Nelson M. E. (2005). Locating the semiotic power of multimodality. Writ. Commun. 22, 224–261. doi: 10.1177/0741088304274170

[ref74] Hurewitz F. Gelman R. Schnitzer B. (2006). Sometimes area counts more than number. Proc. Natl. Acad. Sci. 103, 19599–19604. doi: 10.1073/pnas.0609485103, PMID: 17159143 PMC1748271

[ref75] Hurford J. R. (1975). The Linguistic Theory of Numerals. Cambridge, UK: Cambridge University Press.

[ref76] Jamalian A. Tversky B. (2012). Gestures alter thinking about time. In *Proceedings of the Annual Meeting of the Cognitive Science Society*, p. 34.

[ref77] Kao J. T. Wu J. Y. Bergen L. Goodman N. D. (2014). Nonliteral understanding of number words. Proc. Natl. Acad. Sci. 111, 12002–12007. doi: 10.1073/pnas.1407479111, PMID: 25092304 PMC4143012

[ref78] Kathrada A. Yasseen Y. Varachia Z. (2021). The incidence and quality of graphs in annual reports: a south African analysis of graph disclosure in state-owned entities. Afr. Public Serv. Delivery Performance Rev. 9:11. doi: 10.4102/apsdpr.v9i1.513

[ref79] Keller R. (1998). A Theory of Linguistic Signs. Oxford, UK: Oxford University Press.

[ref80] Kendon A. (2004). Gesture: Visible Action as Utterance. Cambridge, UK: Cambridge University Press.

[ref81] Kendon A. (2014). Semiotic diversity in utterance production and the concept of language. Phil. Trans. R. Soc. B 369:20130293. doi: 10.1098/rstb.2013.0293, PMID: 25092661 PMC4123672

[ref82] Kirk E. Gurney D. Edwards R. Dodimead C. (2015). Handmade memories: the robustness of the gestural misinformation effect in children’s eyewitness interviews. J. Nonverbal Behav. 39, 259–273. doi: 10.1007/s10919-015-0210-z

[ref83] Kita S. (2003). Pointing: Where Language, Culture, and Cognition Meet. Mahwah, NJ: Lawrence Erlbaum.

[ref84] Kitano H. (2004). Biological robustness. Nat. Rev. Genet. 5. doi: 10.1038/nrg1471 15520792

[ref85] Klein E. Nuerk H.-C. Wood G. Knops A. Willmes K. (2009). The exact vs. approximate distinction in numerical cognition may not be exact, but only approximate: how different processes work together in multi-digit addition. Brain Cogn. 69, 369–381. doi: 10.1016/j.bandc.2008.08.031, PMID: 18929439

[ref86] Knops A. Viarouge A. Dehaene S. (2009). Dynamic representations underlying symbolic and nonsymbolic calculation: evidence from the operational momentum effect. Atten. Percept. Psychophys. 71, 803–821. doi: 10.3758/APP.71.4.803, PMID: 19429960

[ref87] Koester A. (2007). “‘About twelve thousand or so’: vagueness in north American and UK offices” in Vague Language Explored. ed. Cutting J. (New York: Palgrave Macmillan), 40–61.

[ref88] Kosara R. (2019a). Evidence for area as the primary visual cue in pie charts. 2019 IEEE Visual. Conf. (VIS), 101–105. doi: 10.1109/VISUAL.2019.8933547

[ref89] Kosara R. (2019b). The impact of distribution and chart type on part-to-whole comparisons. EuroVis (Short Papers), 7–11. doi: 10.2312/evs.20191162

[ref90] Kosslyn S. (2006). Graph design for the eye and mind. Oxford, UK: Oxford University Press.

[ref91] Krawczyk M. (2015). The search for significance: a few peculiarities in the distribution of P values in experimental psychology literature. PLoS One 10:e0127872. doi: 10.1371/journal.pone.0127872, PMID: 26061881 PMC4463849

[ref92] Kress G. Van Leeuwen T. (2020). Reading Images: The Grammar of Visual Design. Abingdon, UK: Routledge.

[ref93] Krifka M. (2007). Approximate Interpretation of Number Words. Berlin: Humboldt-Universität zu Berlin, Philosophische Fakultät II.

[ref94] Lakoff G. Johnson M. (1980). Metaphors We Live By. Chicago, IL: University of Chicago Press.

[ref95] Lakoff G. Núñez R. E. (2000). Where Mathematics Comes From: How the Embodied Mind Brings Mathematics Into Being. New York: Basic Books.

[ref96] Lasersohn P. (1999). Pragmatic halos. Language 75:522. doi: 10.2307/417059

[ref97] Latour B. (1986). Visualization and cognition. Knowl. Soc. 6, 1–40.

[ref98] Lavric E. (2010) “New approaches to hedging,” in Hyperbolic Approximative Numerals in Cross-cultural Comparison. eds. Kaltenböck G. Mihatsch W. Schneider S. (Brill), 123–164.

[ref99] Lewis T. N. Stickles E. (2017). Gestural modality and addressee perspective influence how we reason about time. Cognit. Linguist. 28, 45–76. doi: 10.1515/cog-2015-0137

[ref100] Lindemann O. Abolafia J. M. Girardi G. Bekkering H. (2007). Getting a grip on numbers: numerical magnitude priming in object grasping. J. Exp. Psychol. Hum. Percept. Perform. 33, 1400–1409. doi: 10.1037/0096-1523.33.6.1400 PMID: 18085952

[ref101] Marghetis T. Núñez R. (2013). The motion behind the symbols: a vital role for dynamism in the conceptualization of limits and continuity in expert mathematics. Top. Cogn. Sci. 5, 299–316. doi: 10.1111/tops.12013, PMID: 23460466

[ref102] Marghetis T. Núñez R. Bergen B. K. (2014). Doing arithmetic by hand: hand movements during exact arithmetic reveal systematic, dynamic spatial processing. Q. J. Exp. Psychol. 67, 1579–1596. doi: 10.1080/17470218.2014.897359, PMID: 25051274

[ref103] Mason P. H. Winter B. Grignolio A. (2015). Hidden in plain view: Degeneracy in complex systems. Biosystems 128, 1–8. doi: 10.1016/j.biosystems.2014.12.003, PMID: 25543071

[ref104] McCrink K. Dehaene S. Dehaene-Lambertz G. (2007). Moving along the number line: operational momentum in nonsymbolic arithmetic. Percept. Psychophys. 69, 1324–1333. doi: 10.3758/BF03192949, PMID: 18078224

[ref105] McGlone M. S. Harding J. L. (1998). Back (or forward?) to the future: the role of perspective in temporal language comprehension. J. Exp. Psychol. Learn. Mem. Cogn. 24, 1211–1223. doi: 10.1037/0278-7393.24.5.1211

[ref106] McNeill D. (1992). Hand and Mind: What Gestures Reveal About Thought. Chicago, IL: University of Chicago Press.

[ref107] McNeill D. (2005). Gesture and Thought. Chicago, IL: Chicago University Press.

[ref108] Moxey L. M. Sanford A. J. (1993a). Communicating Quantities: A Psychological Perspective. Mahwah, NJ: Lawrence Erlbaum Associates.

[ref109] Moxey L. M. Sanford A. J. (1993b). Prior expectation and the interpretation of natural language quantifiers. Eur. J. Cogn. Psychol. 5, 73–91. doi: 10.1080/09541449308406515

[ref110] Newstead S. E. Pollard P. Riezebos D. (1987). The effect of set size on the interpretation of quantifiers used in rating scales. Appl. Ergon. 18, 178–182. doi: 10.1016/0003-6870(87)90001-9, PMID: 15676619

[ref111] Nguyen H. A. Hofman J. M. Goldstein D. G. (2022). Round numbers can sharpen cognition. Proc. 2022 CHI Conf. Hum. Factors Comput. Syst. 375, 1–15. doi: 10.1145/3491102.3501852

[ref112] Nicol M. M. Patson N. D. (2022). The effect of gestures on the interpretation of plural references. J. Cogn. Psychol. 34, 454–469. doi: 10.1080/20445911.2021.1998074

[ref113] Nothelfer C. Franconeri S. (2020). Measures of the benefit of direct encoding of data deltas for data pair relation perception. IEEE Trans. Vis. Comput. Graph. 26, 311–320. doi: 10.1109/TVCG.2019.2934801, PMID: 31536003

[ref114] Novack M. Goldin-Meadow S. (2015). Learning from gesture: how our hands change our minds. Educ. Psychol. Rev. 27, 405–412. doi: 10.1007/s10648-015-9325-3, PMID: 26366048 PMC4562024

[ref115] Núñez R. (2004). “Do real numbers really move? Language, thought, and gesture: the embodied cognitive foundations of mathematics” in Embodied Artificial Intelligence. eds. Iida F. Pfeifer R. Steels L. Kuniyoshi Y. (Springer), 54–73.

[ref116] Núñez R. (2008). “A fresh look at the foundations of mathematics: gesture and the psychological reality of conceptual metaphor” in Metaphor and Gesture. eds. Cienki A. Müller C. (John Benjamins), 93–114.

[ref117] Nunez B. L. (2016). The use and misuse of graphs in stand-alone annual reports evidence from Brazilian listed companies. MA thesis. NOVA – School of Business and Economics & Fundaçao Getulio Vargas.

[ref118] Núñez R. Marghetis T. (2015). “Cognitive linguistics and the concept(s) of number” in The Oxford Handbook of Numerical Cognition. eds. Cohen Kadosh R. Dowker A. (Oxford, UK: Oxford University Press), 377–401.

[ref119] Okan Y. Garcia-Retamero R. Galesic M. Cokely E. T. (2012). When higher bars are not larger quantities: on individual differences in the use of spatial information in graph comprehension. Spat. Cogn. Comput. 12, 195–218. doi: 10.1080/13875868.2012.659302

[ref120] Overmann K. A. (2016). The role of materiality in numerical cognition. Quat. Int. 405, 42–51. doi: 10.1016/j.quaint.2015.05.026

[ref121] Overmann K. A. (2018). Constructing a concept of number. J. Numer. Cogn. 4, 464–493. doi: 10.5964/jnc.v4i2.161

[ref122] Padilla L. Castro S. C. Hosseinpour H. (2021). A review of uncertainty visualization errors: working memory as an explanatory theory. Psychol. Learn. Motiv. 74, 275–315. doi: 10.1016/bs.plm.2021.03.001

[ref123] Padilla L. M. Creem-Regehr S. H. Hegarty M. Stefanucci J. K. (2018). Decision making with visualizations: a cognitive framework across disciplines. Cogn. Res. Princ. Implic. 3, 1–25. doi: 10.1186/s41235-018-0120-9, PMID: 30238055 PMC6091269

[ref124] Paivio A. (1971). Imagery and Verbal Processes. Holt, Rinehart and Winston.

[ref125] Paivio A. (1986). Mental Representations: A Dual Coding Approach. New York, NY: Oxford University Press.

[ref126] Pandey A. V. Rall K. Satterthwaite M. L. Nov O. Bertini E. (2015). How deceptive are deceptive visualizations? An empirical analysis of common distortion techniques. In *Proceedings of the 33rd Annual ACM Conference on Human Factors in Computing Systems*, pp. 1469–1478.

[ref127] Parker I. (2001). Absolute powerpoint. New Yorker 28, 76–87.

[ref128] Peirce C. (1955). Philosophical Writings of Peirce. New York, NY: Dover.

[ref129] Perlman M. Clark N. Johansson Falck M. (2015). Iconic prosody in story reading. Cogn. Sci. 39, 1348–1368. doi: 10.1111/cogs.12190, PMID: 25351919

[ref130] Perniss P. (2018). Why we should study multimodal language. Front. Psychol. 9:1109. doi: 10.3389/fpsyg.2018.01109, PMID: 30002643 PMC6032889

[ref131] Peterson L. V. Schramm W. (1954). How accurately are different kinds of graphs read? Audio Vis. Commun. Rev. 2, 178–189. doi: 10.1007/BF02713334

[ref132] Pinhas M. Fischer M. H. (2008). Mental movements without magnitude? A study of spatial biases in symbolic arithmetic. Cognition 109, 408–415. doi: 10.1016/j.cognition.2008.09.003, PMID: 18976986

[ref133] Porter T. M. (1996). “Trust in numbers: the pursuit of objectivity in science and public life” in Trust in Numbers (Princeton, NJ: Princeton University Press)

[ref134] Regier T. Carstensen A. Kemp C. (2016). Languages support efficient communication about the environment: words for snow revisited. PLoS One 11:e0151138. doi: 10.1371/journal.pone.0151138, PMID: 27073981 PMC4830456

[ref135] Rips L. J. (2013). How many is a zillion? Sources of number distortion. J. Exp. Psychol. Learn. Mem. Cogn. 39, 1257–1264. doi: 10.1037/a0031143, PMID: 23244052

[ref136] Ruud P. A. Schunk D. Winter J. K. (2014). Uncertainty causes rounding: an experimental study. Exp. Econ. 17, 391–413. doi: 10.1007/s10683-013-9374-8

[ref137] Shah P. Freedman E. G. (2011). Bar and line graph comprehension: an interaction of top-down and bottom-up processes. Top. Cogn. Sci. 3, 560–578. doi: 10.1111/j.1756-8765.2009.01066.x, PMID: 25164403

[ref138] Shah P. Hoeffner J. (2002). Review of graph comprehension research: implications for instruction. Educ. Psychol. Rev. 14, 47–69. doi: 10.1023/A:1013180410169

[ref139] Simkin D. Hastie R. (1987). An information-processing analysis of graph perception. J. Am. Stat. Assoc. 82, 454–465. doi: 10.1080/01621459.1987.10478448

[ref140] Skau D. Kosara R. (2016). Arcs, angles, or areas: individual data encodings in pie and donut charts. Comput. Graph. Forum 35, 121–130. doi: 10.1111/cgf.12888

[ref141] Smiley C. Plachouras V. Schilder F. Bretz H. Leidner J. Song D. (2016). When to plummet and when to soar: Corpus based verb selection for natural language generation. In *Proceedings of the 9th International Natural Language Generation Conference*, pp. 36–39.

[ref142] Solt S. Cummins C. Palmović M. (2017). The preference for approximation. Int. Rev. Pragmat. 9, 248–268. doi: 10.1163/18773109-00901010

[ref143] Tversky B. (2011). Visualizing thought. Topics in cognitive. Science 3, 499–535. doi: 10.1111/j.1756-8765.2010.01113.x 25164401

[ref144] Ulrike Z. Delgado C. E. E. Dikyuva H. Panda S. De Vos C. (2013). Cardinal numerals in rural sign languages: approaching cross-modal typology. Linguist. Typol. 17, 357–396. doi: 10.1515/lity-2013-0019

[ref145] Van Der Henst J. Carles L. Sperber D. (2002). Truthfulness and relevance in telling the time. Mind Lang. 17, 457–466. doi: 10.1111/1468-0017.00207

[ref146] Walker E. Cooperrider K. (2016). The continuity of metaphor: evidence from temporal gestures. Cogn. Sci. 40, 481–495. doi: 10.1111/cogs.12254, PMID: 26059310

[ref147] Washburne J. N. (1927). An experimental study of various graphic, tabular, and textual methods of presenting quantitative material. J. Educ. Psychol. 18, 361–376. doi: 10.1037/h0074758

[ref148] Winter B. (2014). Spoken language achieves robustness and evolvability by exploiting degeneracy and neutrality. BioEssays 36, 960–967. doi: 10.1002/bies.201400028, PMID: 25088374

[ref149] Winter B. Duffy S. E. (2020). Can co-speech gestures alone carry the mental timeline? J. Exp. Psychol. Learn. Mem. Cogn. 46, 1768–1781. doi: 10.1037/xlm0000836, PMID: 32406722

[ref150] Winter B. Marghetis T. Matlock T. (2015a). Of magnitudes and metaphors: explaining cognitive interactions between space, time, and number. Cortex 64, 209–224. doi: 10.1016/j.cortex.2014.10.015, PMID: 25437376

[ref151] Winter B. Matlock T. Shaki S. Fischer M. H. (2015b). Mental number space in three dimensions. Neurosci. Biobehav. Rev. 57, 209–219. doi: 10.1016/j.neubiorev.2015.09.005, PMID: 26365108

[ref152] Winter B. Perlman M. Majid A. (2018). Vision dominates in perceptual language: English sensory vocabulary is optimized for usage. Cognition 179, 213–220. doi: 10.1016/j.cognition.2018.05.008, PMID: 29966914

[ref153] Winter B. Perlman M. Matlock T. (2013). Using space to talk and gesture about numbers: evidence from the TV news archive. Gesture 13, 377–408. doi: 10.1075/gest.13.3.06win

[ref154] Wood G. Willmes K. Nuerk H.-C. Fischer M. H. (2008). On the cognitive link between space and number: a meta-analysis of the SNARC effect. Psychol. Sci. 50, 489–525.

[ref155] Woodin G. Winter B. Littlemore J. Perlman M. Grieve J. (2023). Large-scale patterns of number use in spoken and written English. Corpus Linguist. Linguist. Theory. doi: 10.1515/cllt-2022-0082 PMC10853912 38344039

[ref156] Woodin G. Winter B. Padilla L. (2022). Conceptual metaphor and graphical convention influence the interpretation of line graphs. IEEE Trans. Vis. Comput. Graph. 28, 1209–1221. doi: 10.1109/TVCG.2021.3088343, PMID: 34110996

[ref157] Woodin G. Winter B. Perlman M. Littlemore J. Matlock T. (2020). “Tiny numbers” are actually tiny: evidence from gestures in the TV news archive. PLoS One 15:e0242142. doi: 10.1371/journal.pone.0242142, PMID: 33201907 PMC7671496

[ref158] Xiong C. Ceja C. R. Ludwig C. J. H. Franconeri S. (2020a). Biased average position estimates in line and bar graphs: underestimation, overestimation, and perceptual pull. IEEE Trans. Vis. Comput. Graph. 26, 301–310. doi: 10.1109/TVCG.2019.2934400, PMID: 31425112

[ref159] Xiong C. Van Weelden L. Franconeri S. (2020b). The curse of knowledge in visual data communication. IEEE Trans. Vis. Comput. Graph. 26, 3051–3062. doi: 10.1109/TVCG.2019.2917689, PMID: 31107654

[ref160] Zacks J. M. Franconeri S. L. (2020). Designing graphs for decision-makers. Policy Insights Behav. Brain Sci. 7, 52–63. doi: 10.1177/2372732219893712

[ref161] Zacks J. Levy E. Tversky B. Schiano D. (2002). “Graphs in print” in Diagrammatic Representation and Reasoning. eds. Anderson M. Meyer B. Olivier P. (Berlin: Springer), 187–206.

[ref162] Zacks J. Tversky B. (1999). Bars and lines: a study of graphic communication. Mem. Cogn. 27, 1073–1079. doi: 10.3758/BF03201236, PMID: 10586582

